# Percutaneous vertebroplasty for SAPHO syndrome with vertebral destruction: a case report and literature review

**DOI:** 10.3389/fmed.2023.1175787

**Published:** 2023-05-09

**Authors:** Yuanpei Cheng, Hao Feng, Junhan Mu, Jialin Chen, Han Wu

**Affiliations:** ^1^Department of Orthopedics, China-Japan Union Hospital of Jilin University, Changchun, China; ^2^Department of Orthopedics Trauma and Sports Medicine, The Affiliated Hospital of Hebei University, Baoding, China

**Keywords:** SAPHO syndrome, osteoarticular lesion, vertebral destruction, percutaneous vertebroplasty, case report

## Abstract

Synovitis, acne, pustulosis, hyperostosis and osteitis (SAPHO) syndrome is a rare musculoskeletal disease characterized by dermatological and osteoarticular lesions. However, SAPHO syndrome is difficult to be diagnosed due to the rarity and complexity. Additionally, there is no standard treatment for SAPHO syndrome based on limited experience. Percutaneous vertebroplasty (PVP) has rarely been reported to treat SAPHO syndrome. We reported a 52-year-old female patient who had a sex-month history of back pain. Palmoplantar pustulosis appeared on the hands and feet. Vertebral destruction was observed on computed tomography (CT) scanning. Laboratory examination showed that erythrocyte sedimentation rate (ESR) and C-reactive protein were elevated. Finally, the patient was diagnosed with SAPHO syndrome and treated with PVP. After the surgery, the back pain was significantly relieved. In this study, we mainly discussed the treatment methods of SAPHO syndrome, and provided a potential treatment for SAPHO syndrome, especially with vertebral destruction, kyphosis, and even pathological fractures.

## Introduction

Synovitis, acne, pustulosis, hyperostosis and osteitis (SAPHO) syndrome, first introduced by Chamot et al. in 1987, is a rare disease characterized by dermatological and osteoarticular lesions (1). The incidence of SAPHO syndrome ranges from 0.0144 per million to 100 per million and is perhaps underestimated due to misdiagnosis (2). Interestingly, SAPHO syndrome predominantly occurs in middle-aged women (3). The etiology of SAPHO syndrome is still unclear, perhaps involving infectious, immunological, and genetic factors (4–7). The clinical symptoms of SAPHO syndrome mainly include dermatological lesions manifesting as palmoplantar pustulosis and severe acne in hands, feet and back, and osteoarticular manifestations presenting as osteitis and hyperostosis in the anterior chest wall, the spine, and the sacroiliac joint (8). The diagnostic criteria of SAPHO syndrome were introduced by Benhamou et al. based on clinical manifestations and radiological examinations (9). SAPHO syndrome could be misdiagnosed as infectious spondylitis, spinal tuberculosis, and tumor on the basis of atypical symptoms. Due to no standard treatment, non-steroidal anti-inflammatory drugs (NSAIDs) are generally considered as the mainstream medications to relieve the symptoms of SAPHO syndrome. Other medications include disease-modifying antirheumatic drugs (DMARDs), corticosteroids, antibiotics, and biologics. In this study, percutaneous vertebroplasty (PVP) as a novel treatment method of SAPHO syndrome is introduced. To the best of knowledge, there have been few studies on the novel treatment method of SAPHO syndrome. The purpose of this study is to introduce a case of SAPHO syndrome treated by PVP, to discuss the treatment methods of SAPHO syndrome, and to provide a potential treatment for SAPHO syndrome, especially with vertebral destruction, kyphosis, and even pathological fractures, which promotes a deeper and more comprehensive understanding of the disease.

## Case presentation

**FIGURE 1:**
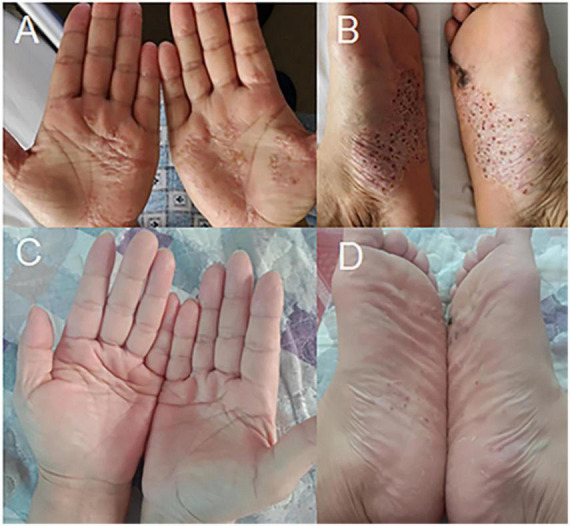
Cutaneous manifestations of SAPHO syndrome **(A–D)**. **(A,B)** Palmoplantar pustulosis was observed on the hands and feet, pre-operatively. **(C,D)** Palmoplantar pustulosis on the hands and feet disappeared during the follow-up period.

**FIGURE 2:**
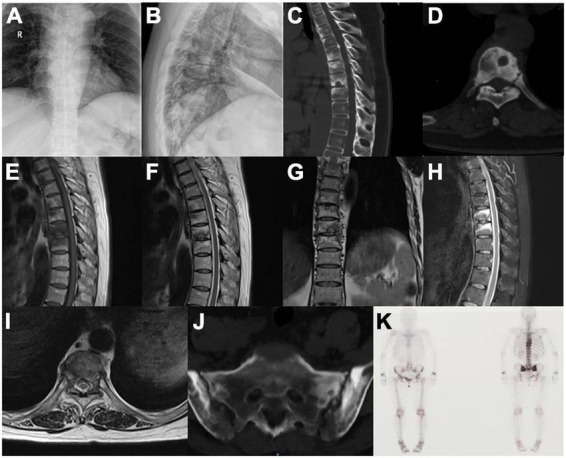
Osteoarticular manifestations of SAPHO syndrome **(A–K)**. **(A,B)** Plain radiographs revealed heterogeneous density of thoracic vertebrae and osteophytes at the edges of thoracic vertebrae. **(C,D)** Thoracic CT scanning demonstrated cortical erosion, osteophyte, and osteosclerosis of thoracic vertebrae. **(E–I)** Thoracic MRI showed thoracic vertebrae with vertebral lesions. **(J)** Sacroiliac joint CT revealed unsmooth joint surface, extensive sclerosis of the bone and patchy low-density shadow. **(K)** Whole-body bone scintigraphy demonstrated multifocal osteoarthritic lesions.

**FIGURE 3:**
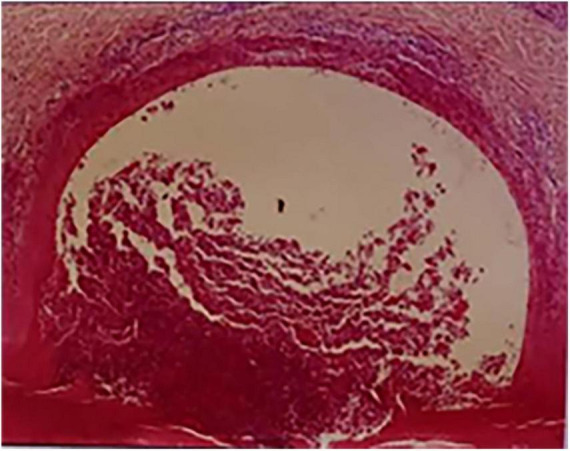
The pathological result showed lymphocyte infiltration.

**FIGURE 4:**
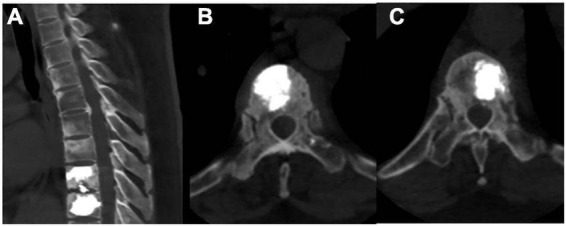
Post-operative radiographic images **(A–C)**. **(A–C)** Post-operative thoracic CT revealed that the bone cement in T8 and T9 vertebrae was well dispersed.

A 52-year-old female patient who had a sex-month history of back pain. The patient took NSAIDs to relieve pain symptoms. However, the patient did not experience significant relief of pain symptoms after taking NSAIDs. More seriously, the patient’s symptoms worsened. The patient failed to walk due to progressive symptoms, and she had to stay in bed due to severe back pain. More treatments were not taken. The patient had difficulty participating in social activities and looking after herself. Therefore, her husband had to take care of her. Eventually, the patient could not tolerate the back pain, and she had to come to our hospital for further treatment. The patient indicated that she had no similar medical history or family history of genetic disorders. The patient also denied the presence of human leukocyte antigen B27 (HLA-B27) in her family history. Physical examination showed back tenderness pain. Palmoplantar pustulosis was observed on the hands and feet (Figures 1A, B). Achilles jerk reflex and knee jerk reflex were normal. The bowel and bladder functions were also normal. Plain radiographs revealed heterogeneous density of some thoracic vertebrae and osteophytes at the edges of some thoracic vertebrae (Figures 2A, B). Thoracic computed tomography (CT) scanning demonstrated cortical erosion, osteophyte, and osteosclerosis of some thoracic vertebrae (Figures 2C, D). The vertebral corner lesion and “kissing” appearance were observed on CT images. Thoracic magnetic resonance imaging (MRI) showed that low signal on T1-weighted images, high signal on T2-weighted images, and high signal on short time of inversion recovery (STIR) images of thoracic vertebrae with vertebral lesions (Figures 2E–I). Sacroiliac joint CT revealed unsmooth joint surface, extensive sclerosis of the bone and patchy low-density shadow (Figure 2J). Multifocal osteoarthritic lesions were found on whole-body bone scintigraphy (WBBS) (Figure 2K). Laboratory examination showed erythrocyte sedimentation rate (ESR) (120 mm/h) and C-reactive protein (10.58 mg/L) were significantly elevated, however, white blood cell count (8.83 × 10^9^/L) and neutrophil percentage (68.8%) were normal. HLA-B27 was positive. No bacteria were found in the bacterial culture. Finally, the patient was diagnosed with SAPHO syndrome based on medical history, clinical symptoms, physical examination, laboratory examination, and radiographic findings. PVP was performed under local anesthesia to treat SAPHO syndrome with vertebral destruction. Puncture needles were placed into the T8 and T9 vertebral body. The puncture location was confirmed with the help of fluoroscopy. Subsequently, the working channel was established. Then, bone cement was slowly injected into the T8 and T9 vertebral body. And the working channel was removed. The pathological result demonstrated that lymphocyte infiltration (Figure 3). Antibiotics were injected intravenously within 24 h after the operation. NSAIDs were administered orally when she felt pain. Post-operative thoracic CT showed that the bone cement in T8 and T9 vertebrae was well dispersed (Figures 4A–C). The patient’s back pain was significantly relieved, post-operatively. On the second day after the surgery, the patient could easily leave the bed and move around. The patient’s symptoms gradually improved during the follow-up period. One year post-operatively, the patient returned to life freely and could participate in social activities. NSAIDs was sometimes taken to relieve pain. Interestingly, the palmoplantar pustulosis on the hands and feet disappeared (Figures 1C, D). Therefore, the patient was satisfied with the treatment.

## Discussion

SAPHO syndrome is an abbreviation of synovitis, acne, pustulosis, hyperostosis, and osteitis, mainly involving dermatological and osteoarticular manifestations. SAPHO syndrome severely impairs the health and life quality of patients, especially those with pathological fractures of the vertebral body (10, 11). Due to the low incidence and the complexity of the symptoms, there is no standard treatment of SAPHO syndrome. We reported a female patient with SAPHO syndrome who treated by PVP.

Due to no specific drug for the treatment of SAPHO syndrome, the aim of its treatment is mainly to alleviate clinical symptoms and delay disease progression. Some treatment methods are listed in Table 1. NSAIDs are considered to be the first-line drugs for pain relief in patients with SAPHO syndrome. Su et al. (12) reported a 59-year-old female patient who was diagnosed with SAPHO syndrome. Antibiotics used to treat SAPHO were ineffective. The patient’s symptoms were relieved after 2 weeks of diclofenac administration. Hagemann et al. (13) described a 57-year-old male patient who suffered from severe pain. The patient’s symptoms were rapidly relieved after ibuprofen administration. However, long-term administration of NSAIDs may produce adverse events such as gastrointestinal side effects and coagulation dysfunction. To the best of our knowledge, DMARDs were applied to treat SAPHO syndrome. Vekic et al. (14) described a 27-year-old male with polyarticular lesions. SAPHO syndrome was diagnosed and treated with methotrexate. After 2 weeks of methotrexate treatment, arthritis improved significantly; however, skin symptoms did not improve. Akcaboy et al. (15) reported an 11-year-old girl with SAPHO syndrome. She was successfully treated with methotrexate. Genovese et al. (16) described 15-year-old boy who presented with SAPHO syndrome. He was treated with methotrexate and achieved good therapeutic effect. However, the efficacy of these drugs still needs further study. Corticosteroids could relieve the dermatological and osteoarticular symptoms of SAPHO syndrome. Wang et al. (17) reported a 31-year-old female patient who had palmoplantar pustulosis and nail lesion. The patient’s symptoms were significantly relieved after 4 weeks of methylprednisolone administration. Xu et al. (18) described a 59-year-old female patient who was diagnosed with SAPHO syndrome. Dermatological and osteoarthritic symptoms improved after prednisone administration. Due to the adverse events, corticosteroids are used as a short-term treatment for SAPHO syndrome (17). Antibiotics are considered for the treatment of patients with SAPHO syndrome, especially those whose bacterial cultures revealed Propionibacterium acnes. Takizawa et al. (19) reported a 63-year-old female who had severe SAPHO syndrome. The symptoms were significantly relieved and remarkable curative effect was achieved after minocycline was taken. In a study of 37 cases of SAPHO syndrome, Assmann et al. (4) found that antibiotics could relieve symptoms during the use of antibiotics; however, symptoms reappeared when antibiotics were discontinued. Tetracyclines, azithromycin, and clindamycin have been reported to treat acne lesions in SAPHO syndrome, but have no efficacy in osteoarthritic manifestations (4). Biological agents are also gradually reported to treat SAPHO syndrome. Yang et al. (20) reported a 44-year-old male with SAPHO syndrome. Anti-inflammatory and analgesic drugs were not very effective. His neck and thoracic back pain improved after using adalimumab for 2 weeks. Palmoplantar pustules also resolved after 4 weeks. Ji et al. (21) reported a 24-year-old woman who had a 1-year history of cutaneous manifestations with palmoplantar pustules and pain in the sternoclavicular joint. Methotrexate and cyclosporin had poor therapeutic effects. Palmoplantar pustules were significantly relieved, and joint pain was almost completely relieved after 4 weeks of secukinumab administration. The cutaneous lesions were completely relieved after 3 months. No adverse events were observed during the follow-up period. Adalimumab relieved clinical symptoms in patients with SAPHO syndrome, however, it did not slow the progression of osteoarthritic lesions (22). Long-term efficacy still needs further validation. Li et al. (11) described a 29-year-old female who had the right sternoclavicular joint and back pain and cutaneous lesions. A pathological fracture of the vertebral body was detected and was fixed with a thoracolumbar brace. However, the underlying kyphoscoliosis deformity cannot be managed. Nakamae et al. (23) reported a 73-year-old male who had palmoplantar pustulosis and low back and leg pain. L3 vertebral destruction was observed on CT scanning. Fixation surgery were performed to treat SAPHO syndrome. The pain symptoms were significantly relieved after an operation. In our study, PVP was performed to treat SAPHO syndrome due to vertebral destruction. PVP is a minimally invasive procedure that has the advantages of minimal surgical trauma and short operating time. PKP could restore the height of the vertebrae and prevent the development of kyphosis and pathological fractures. The heat generated during the PVP procedure could destroy inflammatory factors and peripheral nerves to relieve pain. PVP may be a better surgical option than internal fixation surgery for SAPHO syndrome with vertebral destruction. Future patients, especially those with vertebral destruction, may choose PVP as an alternative treatment option for SAPHO syndrome.

**TABLE 1:** Summary of treatment methods for SAPHO syndrome.

Categories	Treatment method	Outcome	Research type	References
NSAIDs	Diclofenac	Improvement	Case report	Su et al. (12)
	Ibuprofen	Improvement	Case report	Hagemann et al. (13)
DMARDs	Methotrexate	Improvement	Case report	Vekic et al. (14)
	Methotrexate	Improvement	Case report	Akcaboy et al. (15)
	Methotrexate	Improvement	Case report	Genovese et al. (16)
Corticosteroids	Methylprednisolone	Improvement	Case report	Wang et al. (17)
	Prednisone	Improvement	Case report	Xu et al. (18)
Antibiotics	Minocycline	Improvement	Case report	Takizawa et al. (19)
	Azithromycin + doxycycline	Improvement	Retrospective study	Assmann et al. (4)
Biologics	Adalimumab	Improvement	Case report	Yang et al. (20)
	Secukinumab	Improvement	Case report	Ji et al. (21)
	Adalimumab	Improvement	Case report	Garcovich et al. (22)
	Leflunomide	Improvement	Case report	Scarpato and Tirri (24)
Surgery	Fixation surgery	Improvement	Case report	Nakamae et al. (23)

However, only one patient with SAPHO syndrome was included in this study. Further studies with large sample size are still needed to explore the efficacy of PVP in the treatment of SAPHO syndrome.

## Conclusion

SAPHO syndrome is a rare musculoskeletal disease characterized by dermatological and osteoarticular lesions. SAPHO syndrome is difficult to be diagnosed due to the rarity and complexity. There is no standard treatment for SAPHO syndrome based on limited experience. In this study, PVP was performed to treat SAPHO syndrome with vertebral destruction after the failure of NSAIDs therapy. In order to avoid the occurrence of kyphosis and pathological vertebral fractures, orthopedic surgeons should carefully evaluate the condition of patients with SAPHO syndrome and determine the best surgical treatment plan. PVP may be a better surgical option for SAPHO syndrome, especially for patients with vertebral destruction.

## Data availability statement

The raw data supporting the conclusions of this article will be made available by the authors, without undue reservation.

## Ethics statement

The studies involving human participants were reviewed and approved by the Ethics Committee of China-Japan Union Hospital of Jilin University. The patients/participants provided their written informed consent to participate in this study. Written informed consent was obtained from the patient for the publication of any potentially identifiable images or data included in this article.

## Author contributions

YC and HW contributed to the conception and design of the study and revised the manuscript. YC collected the clinical data and wrote the manuscript. HF designed the figures and provided the valuable comments. JM and JC contributed to the literature search and provided the valuable comments. All authors contributed to the article and approved the submitted version.
